# Euro Banknote Recognition System for Blind People

**DOI:** 10.3390/s17010184

**Published:** 2017-01-20

**Authors:** Larisa Dunai Dunai, Mónica Chillarón Pérez, Guillermo Peris-Fajarnés, Ismael Lengua Lengua

**Affiliations:** 1 Research Center in Graphic Technology, Universitat Politècnica de València, Camino de Vera s/n, 5L, Valencia 46022, Spain; gperis@upv.es (G.P.-F.); ilengua@degi.upv.es (I.L.L.); 2 Department DSIC, Universitat Politecnica de Valencia, Valencia 46022, Spain; monica.chp@gmail.com

**Keywords:** blindness, image processing, object detection, object recognition, banknote currency

## Abstract

This paper presents the development of a portable system with the aim of allowing blind people to detect and recognize Euro banknotes. The developed device is based on a Raspberry Pi electronic instrument and a Raspberry Pi camera, Pi NoIR (No Infrared filter) dotted with additional infrared light, which is embedded into a pair of sunglasses that permit blind and visually impaired people to independently handle Euro banknotes, especially when receiving their cash back when shopping. The banknote detection is based on the modified Viola and Jones algorithms, while the banknote value recognition relies on the Speed Up Robust Features (SURF) technique. The accuracies of banknote detection and banknote value recognition are 84% and 97.5%, respectively.

## 1. Introduction

Across the world, there are approximately 285 million people with visual disabilities; of those, 39 million of them are blind. Out of the 39 million blind people, 5.9 million live in Africa, 3.2 million in America, and 2 million in Europe [1]. It is also noted that 80% of blind people live in developing countries. Sight is a primary skill for the existence of human beings. It is a privilege to see around us, allowing us to perform daily tasks. Vision is not only a skill that helps activities, but also influences the behavior of human beings. Blindness affects a number of factors in the psychological behavior of people; a blind person is more likely to have depression than a person with impaired vision or a person with normal vision [2]. They are also more likely to suffer from anxiety [3], a lack of social relationships [4], etc.

One of the most significant problems blind people experience—apart from mobility and navigation tasks—is the identification and location of objects in space. The ability to detect, locate and recognize objects helps humans to meet their need for security and to trust their environment. One of the problems usually encountered by blind people is the recognition of the value of banknotes. The official currency used in the countries of the Eurozone is the Euro, and it is the second most traded currency in the world after the US dollar. The Euro banknotes are issued in denominations of €5, €10, €20, €50, €100, €200, and €500. According to the European Central Bank, the front of the banknotes is characterized by designs that reflect the most representative architectural styles of seven periods of European history [5]. Doors and windows symbolize openness and cooperation on the continent, and the bridge on the reverse side depicts the close cooperation between Europe and the rest of the world. The name in the Euro and the European Central Bank represents the 11 official languages in the EU as of 2002. The EU flag contains 12 stars, indicating the dynamism and harmony among the countries of the Eurozone. The European Central Bank introduced the Look-Feel-Tilt design in order to differentiate the value of Euro banknotes. Other differentiating features include size, color, texture, security threads, watermarks, holographic bands, iridescent band, holograms, and inks varying in color. It is noted that the Euro banknote designs were developed in collaboration with the European Blind Union. In 1997, Klatzky and Lederman demonstrated that touch is one of the more efficient methods of object detection [6]. 

Currently, in order to be able to identify the banknotes, many blind users distribute them in advance, by value, into different pockets; this allows them to know the amount they are carrying. However, this classification requires qualifying time or a third person to help them. The artificial intelligence improvements and development of technologies have enabled great advances in the use of artificial vision for the recognition of the value of several currencies, such as Euro banknotes [7,8,9], Dollars [10,11,12,13], Rupees [14], Mexican banknotes [15], the currency of Saudi Arabia [16], etc.

Most of the work in banknote recognition is based on neural networks [15], Markov models, Principal Component Analysis (PCA) [13], or a Speed Up Robust Features (SURF) model [17].

This paper describes a system for the detection and recognition of Euro banknotes based on the application of Haar techniques proposed by Viola and Jones [18] and SURF [18]. The Haar features have been used for the detection of banknotes, and the SURF technique when identifying the banknote value. The Haar features are employed in order to identify the zone of interest in the image, instead of analyzing each pixel. This method allows for a drastically reduced computational time. By using Haar features, a set of local features is extracted afterwards, which are classified with the AdaBoost algorithm. The aim of the use of AdaBoost algorithms is to distinguish the Euro banknotes from complex images. The AdaBoost algorithm assigns a weight to each sample, and selects the feature that best classifies the sample according to the weight. Once the banknote is detected, the Speed-Up Robust Features algorithms are used in order to detect interest points in an image, each with their own characteristics. SURF algorithms use integral images, as well as the algorithms used on the banknote detection, which drastically reduces the computational time. The points of interest are detected by using the Fast-Hessian matrix. It describes the intensity content within the point of interest compared with neighboring items. Once the information of interest points and the neighboring items are recognized, the SURF descriptor is extracted from the region. Finally, features are matched between the trained image and the image acquired by the system.

The paper is organized as follows: Section 2 enumerates the materials used on the prototype development. Section 3 describes the method of classification and detection based on Haar feature extraction, and the method of banknote recognition based on SURF methodology is also described. Section 4 summarizes the experiments and the results. Section 5 presents the conclusions of the work. 

## 2. Materials

**Figure 1:**
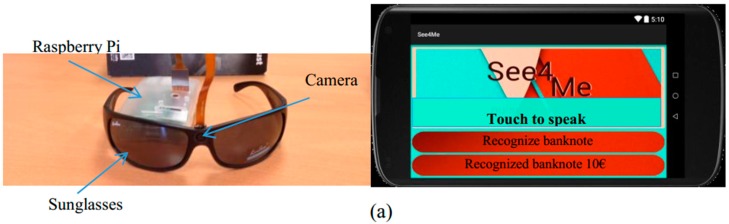

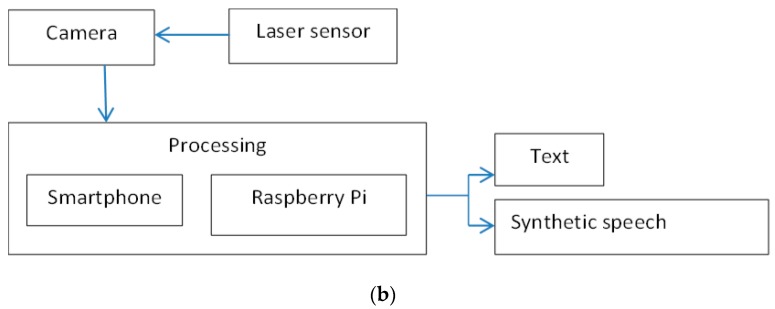
(**a**) System hardware and graphic user interface. The micro camera connected to the Raspberry Pi hardware is embedded in the glasses; (**b**) Schema of the hardware prototype.

For the development of the system for banknote recognition, the following equipment was used: a Raspberry Pi 2 B microcomputer of credit card size (12, 7 × 10, 2 × 7, 6 cm), a Raspberry Pi No Infrared (NoIR) camera, a smartphone, and a pair of sunglasses, as shown in Figure 1. The images are taken with the Raspberry Pi NoIR camera that has integrated an infrared LED. The images captured in real time by the camera are processed by the Raspberry Pi 2 B microcomputer. Through voice commands, the system initializes the image acquisition, afterward realizes the image processing and the banknote recognition algorithms described in Section 3, and returns the name of the recognized banknote through a textual and synthetic speech on the smartphone; see Figure 1b.

The Raspberry Pi 2 B instrument is a microcomputer with a 900 MHz quad-core processor, ARM Cortex, A7 CPU, 1 GB RAM memory, and a Micro SD card with a memory of 32 GB. The ARMv7 processor operates on GNU/LINUX, as well as on Microsoft Windows 10. The graphic video processor is a VideoCore IV that allows fast and great precision on image processing. The Raspberry Pi 2 B is a low-cost, small electronic instrument that is good for image processing. 

The 5 MP Raspberry Pi NoIR is a micro camera sized at 25 × 24 × 9 mm, weighs over 3 g, and has an OMNIVISION 5647 sensor. It has a fixed focus, and can be embedded in a pair of sunglasses. The camera is directly connected to the BCM2835 processor through a CSI bus that makes the processing time shorter. The camera is able to work in the darkness due to the "near-infrared lighting" ability—1000 nm approximately 800 nm wavelength light.

The smartphone selected for the device is an Android-based Samsung Galaxy. For iteration with the Android device, a Bluetooth server with the command “recognize banknote” was created. The application system is solely guided via voice; however, the response is given both via voice and visually shown on the mobile screen (Figure 1).

The interface is composed of a button element that occupies ¾ of the screen so that the iteration is not complicated, and two TextView elements. One of the text elements is used for the voice recognition command, while the second text element presents the recognition output. The speech synthesizer reads both recognition results.

The sunglasses were designed, modeled, and printed with a 3D printer. The implementation of the processing unit is based on Python 2, a Raspbian programming language, the Open CV library, and the Android Studio used for the development of the mobile interface application and the communication algorithms.

## 3. Methods

**Figure 2:**
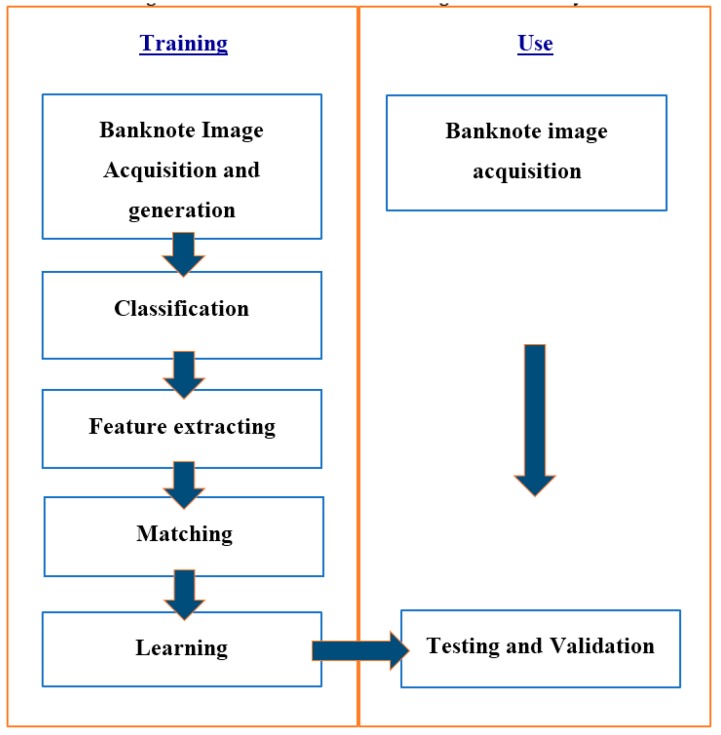
Block diagram of the proposed method.

The Euro banknote detection and recognition system consists of two operation blocks: the training and the real-time use. Figure 2 describes the block diagram of the system functionality. 

The Haar features and AdaBoost algorithms proposed by Viola and Jones [18] are the core of the algorithm for the Euro banknote detection, while the Fast-Hessian matrix is the core of the SURF descriptor [19] that is used for detection of points of interest between images and matching them. 

### 3.1. Euro Banknote Detection 

Euro banknote detection algorithms are based on Haar features proposed by Viola and Jones [17]. The method is based on the classification of the integral image, and not by analyzing each image pixel, which significantly reduces the processing time. Likewise, the method used to encode the characteristics common to all objects to be detected is from a set of training images. The characteristics of Haar type based on the Haar functions presented by Papageorgiou [20] analyze the finite number of rectangles applied to a rectangle of a gray-scale image.

The Viola and Jones method detects the edges in order to identify areas of interest by analyzing the two rectangles. It also detects the lines and analyzes the four rectangles, and detects shapes by analyzing the three rectangles.

Taking the algorithm proposed by Viola and Jones as a basis, we applied a series of modifications proposed by Liendhart and Maydt [21]. Such modifications are based on the application to Haar characteristics: a 45° rotation and scaling. This modification enables a 10% increase by the hit rate detector.

**Figure 3:**
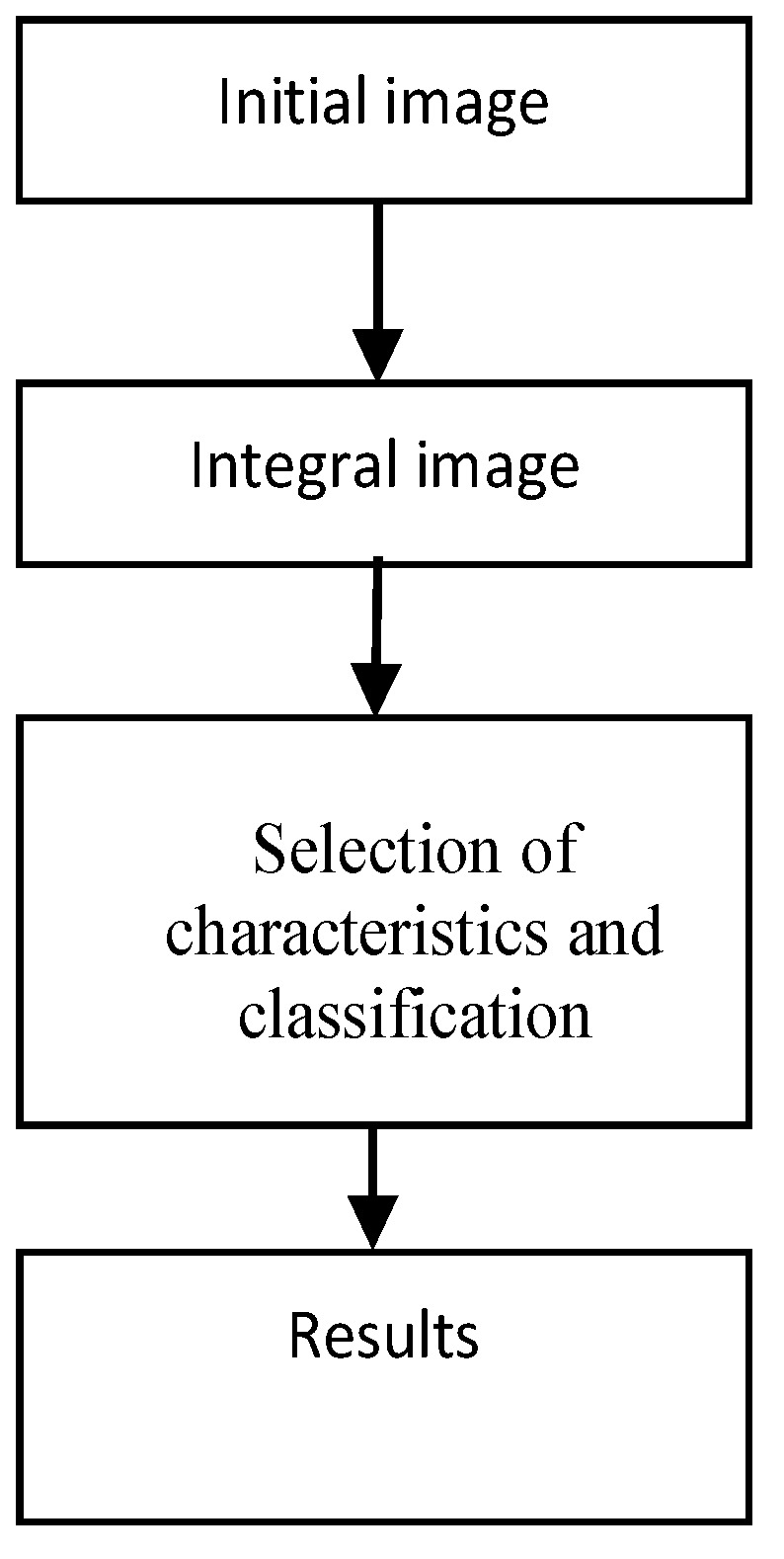
Block diagram of the detection process.

The banknote detection process requires the image processing of the initial input image that defines the overall image, and selects features for classification. In Figure 3, an overview of the detection process is shown. 

#### 3.1.1. Integral Image 

The processing time of all pixels in all regions involves a high computational cost, which slows down the detection process. To avoid these costs, we applied the computational methods proposed by Viola and Jones, focused on integral analysis of images instead of gray values.

Let *ii* be an integral image with the same size as the original image *i*, where *x*, *y* represents the coordinates of each pixel of the integral image, assuming that (0.0) represents the upper left corner of the image and *i*(*x*,*y*) is the original image.

(1) $$ii\left(x,y\right)=\underset{x^{\prime }<x,\;y^{\prime }<y}{\sum }i\left(x^{\prime },y^{\prime }\right)$$

(2) s(x,y)=s(x,y−1)+i(x,y)ii(x,y)=ii(x−1, y)+s(x,y)

The value *s*(*x*,*y*) is the region with the cumulative sum. 

#### 3.1.2. Selection of Characteristics and Classification

**Figure 4:**
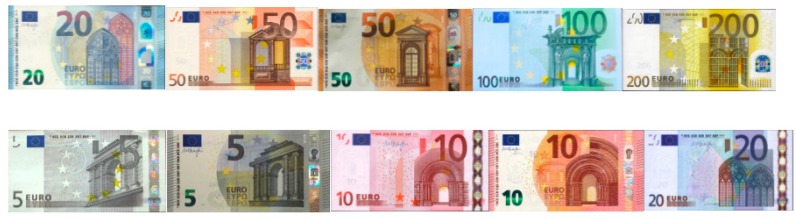
Positive samples: Pictures taken from real Euro banknotes, both old and new issue.

**Figure 5:**
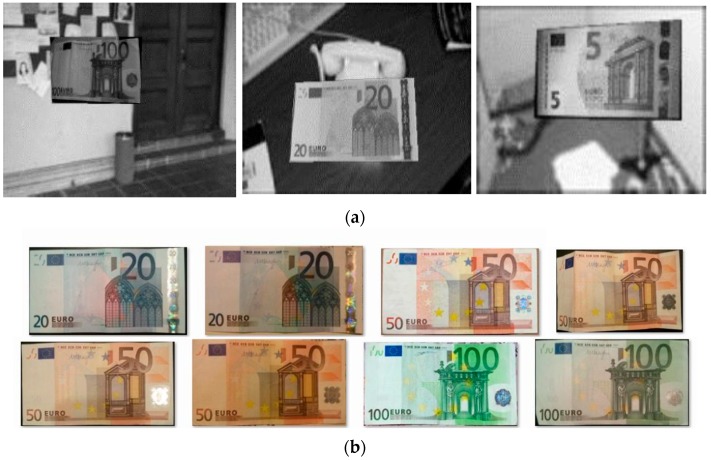
Training samples: Samples of real Euro banknotes and banknotes with background exposed. (**a**) Real euro banknotes with background; (**b**) Real euro banknote pictures.

A set of 200 sample images named positives was taken with the Raspberry Pi-NoIR camera, as shown in Figure 4. The negative images were 452 generic images extracted from the Background Image Dataset developed by Weber [22]. These images contain images of the indoor and outdoor environment around the Caltech campus and the Vision Lab of the California Institute of Technology. Afterwards, adding negative background and rotation have modified the positive images creating 2000 training samples, as shown in Figure 5. In order to obtain a fast processing time, the image size was defined as 80 × 40 pixels. 

The boosting algorithm called Gentle AdaBoost was used to select the features for the training. This algorithm is used to integrate small and simple classifier cascade, since the Haar characteristics have different sizes depending on the size of the rectangles. In this case, the algorithms progressively eliminate sub-windows with negative images in each iteration. The cascade classification avoids a huge number of sub-windows that do not fit with the image of the banknote. In an image of 24 × 24 pixels, over 180,000 different Haar features can be applied.

This means that the Gentle AdaBoost algorithm is looking for a small number of positive characteristics that do not have a significant variation. Thus, it assigns weights to each selected sample and selects the characteristic that best classifies samples based on the weights. 

The weight is calculated as:

(3) $$\theta =\frac{1}{2}\ln\left(\frac{1-\epsilon_{m}}{\epsilon_{m}}\right)$$

where $\epsilon_{m}$ is the error rate, and is calculated as:

(4) $$\epsilon_{m}=\frac{\sum_{y_{i}=k_{m}\left(x_{i}\right)}w_{i}^{\left(m\right)}}{\sum_{i=1}^{N}w_{i}^{\left(m\right)}}$$

where *x* and *y* are the example images, *m* represents the number of iterations, *k* is the weak classifiers, and the $w_{i}$ are the weights.

The weight θ becomes greater when $\epsilon_{m}$ gets smaller.

A classifier of 25 stages was employed, where each stage had a hit rate of 99.9%, and a false positive rate of 50% for the training process system.

Given the set of features and a set of training, and by using the positive images and negative images, the system has to perform the banknote detection tasks.

### 3.2. Banknote Recognition Algorithms

For banknote recognition, the SURF technique is employed. Using this technique, previously detected banknotes (using several samples of each type) were compared, and the most similar is determined.

**Figure 6:**
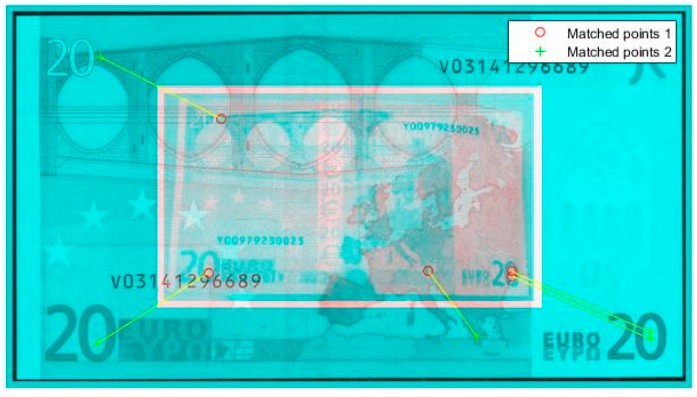
Points of interest matching with Speed Up Robust Features (SURF) method: experiment during the training period with €20 banknote.

All samples were counted by removing their attractions and descriptors and saving into a file for future access for recognition process, as shown in Figure 6. For the recognition process, it is important to obtain the points of interest of the samples.

To perform the extraction of the points of interest and local value changes around the point, the algorithm uses a detector blob based on the Hessian matrix. That determinant is also used to select the scale and the minimum threshold of the determinant value of the Hessian matrix. It allows the computation of the interest points with the threshold higher than the calculated value.

Given a point *p*(*x*, *y*) of the integral image *I*, the Hessian matrix *H* (*p*, *σ*) is calculated at the point p with σ scale, where *L_xx_*(*p*, *σ*) to *L_yy_*(*p*, *σ*) values are those derived from second grade grayscale imaging.

(5) $$H\left(p,\sigma \right)=\left(\begin{array}{c} L_{xx}\left(p,\sigma \right)\;\;\;L_{xy}\left(p,\sigma \right)\\ L_{xy}\left(p,\sigma \right)\;L_{yy}\left(p,\sigma \right) \end{array}\right)$$

*L_xx_(p, σ)* is the convolution of the partial derivative of second degree of Gaussian $\frac{\partial^{2}}{\partial x^{2}}g(\sigma )$ with the integral image *I* at the point *p*. The same method is applied for *L_yy_(p, σ)* and *L_xy_(p, σ).*

Since the points of interest must be found at different scales, filter boxes of different sizes allow a quick evaluation of the partial derivatives of second-degree Gaussian to expand.

For rotation descriptor neighboring areas identified in the dominant orientation, a Haar wavelet filter is applied.

The scale space is divided into a number of a series of responses that cover doubling of scale. In order to obtain the lowest scale space σ an output of *9 × 9* filters are used. The filters are applied iteratively from the higher size to the smallest one, calculating several level of the H determinant.

(6) $$\det\left(H^{t}\right)\approx D_{xx}^{t}D_{yy}^{t}-{\left(wD_{xy}^{t}\right)}^{2}$$

where $D_{xx}^{t}$, $D_{yy}^{t}$, and $D_{xy}^{t}$ are the approximations for the second-order Gaussian partial derivative in *x*, *y*, and *xy* directions, respectively. The *w* represents the relative weight of the filter responses.

Once the filters are obtained, they are normalized with respect to the mask size.

Ergo, the location of the points of interest in the space scales is determined by applying the Hessian matrix 3 × 3 × 3 neighborhood. The operation finds the maximum interpolated later in scale and image space. 

Once the points of interest are calculated, a descriptor is used in order to better describe the point of interest, introducing the lighting distribution of the pixels of the *n* neighbors that surround the point of interest. Then, the point of interest is described for a window of 20 and divided into 4 × 4 regions. For each subregion (all 4 × 4), the vector v is calculated:

(7) v=(∑dx, ∑dy, ∑|dx|, ∑|dy|)

Finally, finished banknote recognition matches are achieved by applying the two images. Fast Library for Approximate Nearest Neighbors (FLANN) was used to determine the points of convergence. The library contains a set of algorithms that work best for nearest neighbor search, meaning that the algorithms determine the parameters that give the best solution [23]. The algorithm for fast nearest neighbor search is based on the structure of the dataset and the desired search precision. These algorithms use the hierarchical *k*-means three and *kd*-threes as well as the automatic selection of the optimal algorithm. The hierarchical *k*-means three algorithm divides the dataset at each level into *K* clusters. It uses the classic k-means three that randomly selects *k* data points as initial means. The *k*-means algorithm is used in order to set the *k* data points to two, in order to divide the dataset into two subsets. Then, the two subsets are divided again into two subsets by setting *k* to two. The k-means algorithm is based on the Euclidean distance. The *kd*-three is a data structure that stores a finite set of points from *k*-dimensional space. This algorithm is efficient for low dimensions and rapidly decreases its performance for high-dimension spaces. Finally, the automatic selection of the optimal algorithm is used. The algorithm for fast nearest neighbor search is based on the structure of the dataset and the desired search precision. The automatic selection determines the parameters that give the best solution from both algorithms.

Technical features optimized for high-dimensionality to make the matching *k*-nearest neighbors or k-means clustering were used. The *k* points of interest of the image detected are selected for each point of interest of the sample, ordered by less distance. In this case, two matches are obtained for each point of interest, from which one is supposed to be eliminated. For eliminating matches, the ratio of Lowe [24]—which determines whether the ratio of distance between a pair of points is greater than 0.8—is used. With the Lowe ratio, matches that have a distance higher than 0.8 are eliminated, as a candidate that presents more false matches.

Once the algorithm is defined, the original image taken with the Raspberry Pi NoIR camera is compared with each of the 2000 samples. For this, the descriptors and the points of interest are defined between both images. Afterwards, the matching of the points of interest is used by applying k-nearest neighbours. In our case, the *k* = 2 hat means that the matching is realized with two images with two neighbours. So, each point of interest obtains two candidates, and one of them is eliminated. The eliminated one is the point of interest whose distance rates is higher than the Lowe rate defined as 0.8. The ratio of points of interest that coincide with the original image is measured. The sample whose coincidence is greater is selected, and the name of the respective sample is returned. 

In Figure 6, the results of the recognition of a €20 banknote is presented by using SURF recognition algorithms. The blue image is the training sample, and the white transparent image in the center is the image taken with the Raspberry Pi NoIR camera of the €20 banknote. Red dots represent the matched points between both images.

During the training period, the processing time was also measured. The training hardware used was the Raspberry Pi 2 B model with the CPU ARM 1176JZF-S with 700 MHz and 512 MB of RAM memory. The operating system is Raspbian. The dimensions of the images were 80 × 40 pixels in order to reduce processing time. Initial images from the camera have 2592 × 1944 pixels, requiring more processing time. From these motifs on the image acquisition algorithms, the images are resized to 80 × 40 pixels. In our case, the processing time for the training period depends on the number of new images introduced for the training. During the training period, the system uses 99% of the system processing capacity and over 6 min for the training. 

## 4. Experiments and Results

### 4.1. Banknote Detection Results

Once the positive and negative images and the samples have been obtained and the training phase developed, we proceed to the banknote detection process. We perform several iterations in which the image scanning, image scaling, or resizing of the window are made. Python was used in order to process the whole process of banknote detection. Two thousand positive images and 2000 negative images were tested for banknote detection. 

The detection was performed for images of 80 × 40 pixels. Then, the Python code was used to load the sorter, capture images, and detect banknotes.

During the detection process, the results depend on the *minNeighbors* and *scaleFactor* parameters. The *minNeighbors* parameter plays the role of the threshold, indicating the number of neighbors that the rectangle of the banknote should have. The *scaleFactor* represents the reduction ratio of the image in each iteration during the detection process.

**Figure 7:**
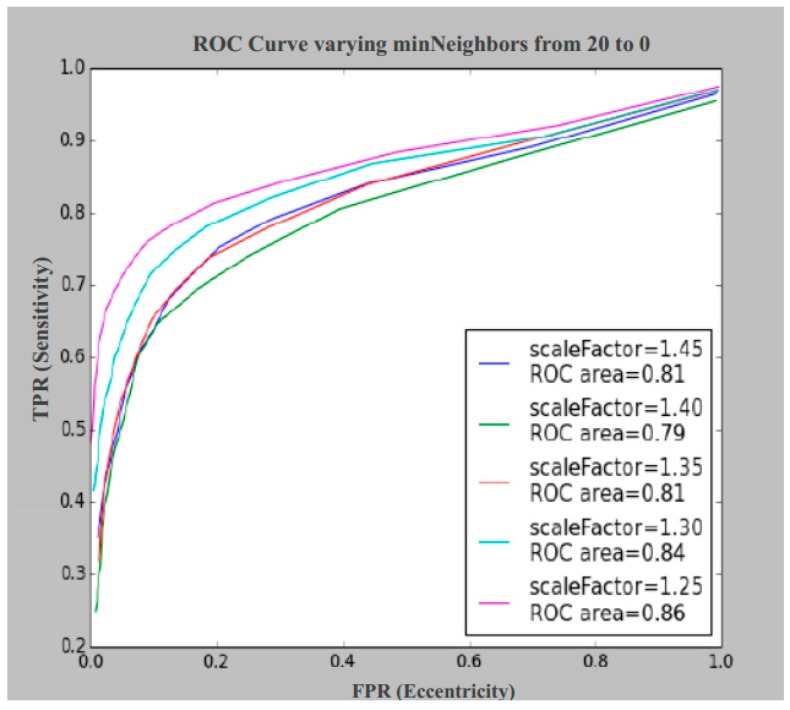
ROC (Receiver Operating Characteristic) curve for the Euro banknote for scale Factor = 1.45, scale Factor = 1.40, scale Factor = 1.35, scale Factor = 1.30 and scale Factor = 1.25. FPR: false positive rate; TPR: true positive rate.

In Figure 7, the ROC (Receiver Operating Characteristic) curves representing the results are displayed for different values of *scaleFactor* by varying the parameter *minNeighbors* from 20 to 0.

To generate the ROC curve, hit rates of false positives (FPR) and the true positive rate (TPR) were calculated by:

(8) $$TPR=\frac{VP}{\left(VP+FN\right)}\;FPR=\frac{FP}{\left(FP+VN\right)}\;TA=\frac{VP+VN}{\left(VP+VN+FP+FN\right)}$$

The value VP represents true positives, FP represents false positives, FN represents false negatives, and VN represents true negatives.

As can be seen, the scale factor values are higher than 1.25, because with lower values, it generates false positives and does not improve the results. 

**Figure 8:**
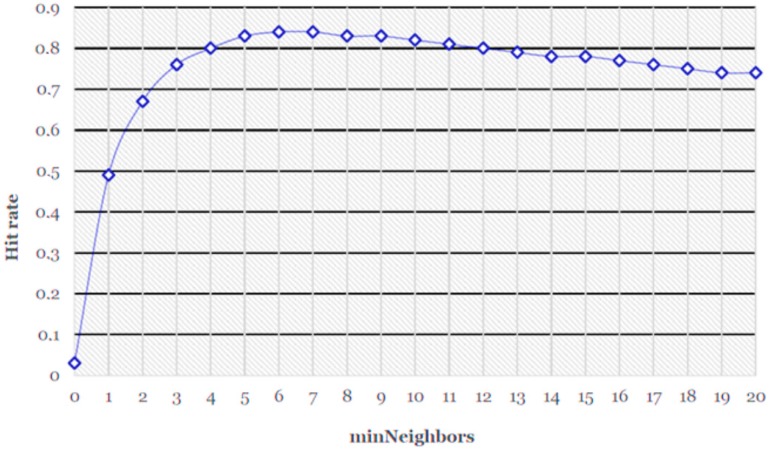
Hit rate with the scale factor (SF) = 1.25: the best values are between the hit rates 0.8 and 0.9.

The average value of the hit rate is 0.736; i.e., 73.6% of the samples correctly classified according to Equation (8). Regarding Figure 7 and Figure 8, the *minNeighbor*s can be fixed to 6, where the best hit rates are obtained graphically between 0.8 and 0.9. With these data we set the value *scaleFactor* = 1.25 and *minNeighbors* = 6, obtaining the hit rate at 0.84, or 84%.

**Figure 9:**
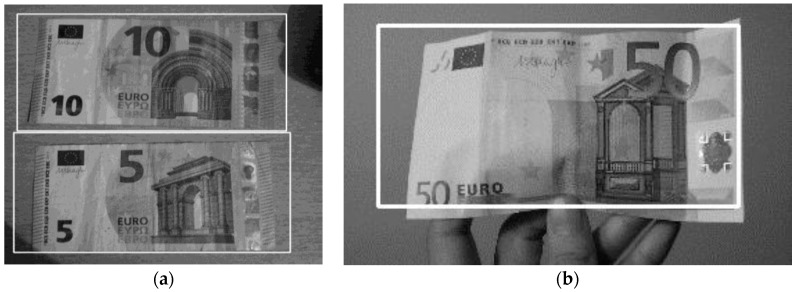
Banknote detection examples: white rectangle is generated around the banknote once it is detected. (**a**) Banknote detection from a surface; (**b**) Banknote detection on the human hand.

In Figure 9, the results of banknote detection of the €5, €10, and €50 banknotes are shown. In Table 1, the banknote detection matrix of confusion is represented.

### 4.2. Banknote Recognition Results

Two hundred photographs of the €5, €10, €20, €50, and €100 banknotes were taken for banknote recognition. By applying the recognition algorithm, a 97.5% HR (Hit Rate) was obtained. Table 2 reports the result of the hit rate. The hit rate is calculated as:

(9) HR=success/(success+false)

The system has been experimented in both 119 tests in an indoor environment and 50 tests in an outdoor environment under a lighted environment and darkness with normal vision and blind users. During the experiments, €5, €10, €20, and €50 banknotes were tested at different distances. The minimum available distance is 20 cm, and the maximum is 60 cm. 

Table 3 reports the results of real Euro banknote recognition tests in illuminated indoor environment for a distance of 45 cm between the camera and the banknote. A set of tests has been done in the same environment in darkness. The results demonstrated that the system obtained a 97.6% degree of accuracy.

For €10 banknotes, the system recognized “not recognized banknote” at a rate of 4%. For the distance of 38 cm. in an illuminated environment, the system had an accuracy of 95.58%. The results are reported in Table 4. 

**Figure 10:**
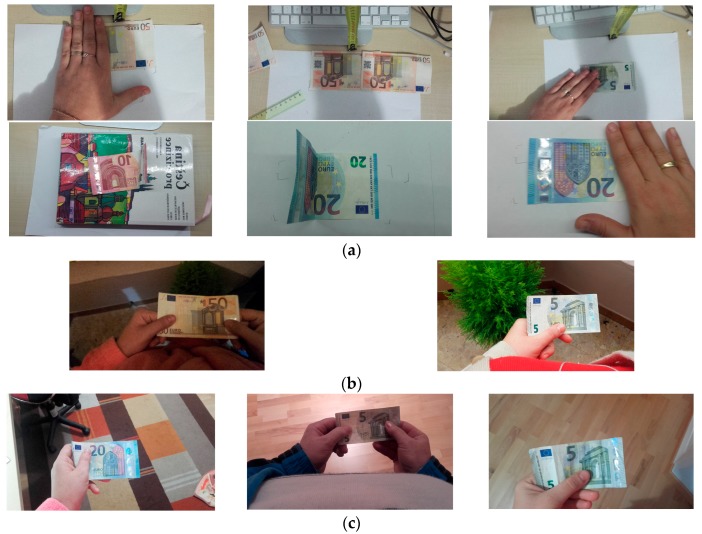
Folded and covered Euro banknotes in indoor and outdoor environment cases used in trials: (**a**) Cases where real banknotes are on the table; (**b**) Real banknotes held by the users—in this case, the environment is an open environment, both night and day; (**c**) The banknotes are held by the user in an enclosed environment (at home and in a shop with artificial illumination).

**Figure 11:**
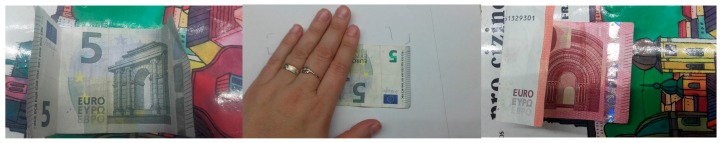
Crumpled and folded Euro banknotes: false recognition cases.

A set of tests has been done for €5 and €50 banknotes where the banknote is wrinkled, where half of the banknote is covered by a hand, and where the banknote is accompanied by different objects, as shown in Figure 10. In the case of the €5 banknotes, the system accuracy was 69.23%. The most responses of “The banknote has not been recognized” were obtained in the case when the banknote was almost doubled. Examples of error cases are shown in Figure 11.

For the €50 banknotes, the system accuracy was 100% in all cases. 

We also measured the processing time for the recognition method. The order command varied between 2 and 3 seconds, and the processing time varied from 9 to 13 seconds. The system was supposed to have done more than 50 tests consecutively. After ten consecutive orders, the recognition time starts to increase. These phenomena occur due to the Bluetooth communication between the mobile application and the processing hardware, and due to the processing hardware. When the system gets collapsed, the answer is “The Raspberry Pi is out of function”, “The order is not recognized”, or “Any banknote is recognized”. In addition, we have to mention that in real conditions, the users use banknote recognition in several situations: when preparing to go shopping, when counting the returned amount following a shopping transaction, or in the bank. From this point of view, it is less probable that the system collapses.

## 5. Conclusions

This paper deals with the hardware and software development of a portable and effective intelligent system for Euro banknote detection and value recognition for blind people. Due to the high number of requirements of the blind people for an easy and effective system for everyday usage, the proposed development uses simple and cheap electronics; i.e., Raspberry Pi, Raspberry Pi NoIR camera communicating with an Android based smartphone, and open software such as Open CV and synthetic speech. Due to the infrared lasers of the camera, the system is able to work both in no or reduced illumination or in dark environments. Due to the training algorithm, they make the system more robust and effective. The tests were performed on real Euro banknotes with users that have normal vision and blind users. The detection hit rate was 84%, while the recognition hit rate was 97.5%. For crumpled banknotes, the recognition accuracy was 69.25%. Differences between the darkness and lighting environment on banknote recognition were perceived, due to the camera quality. The total processing time for training was between 3 min and 7 min, depending on the quantity of new pictures of the banknotes that were introduced. The training processing time is also influenced by the image size used by the system for processing and by the hardware characteristics. The mean banknote recognition time was 11 s.

Future work will deal with the improvement of the detection and recognition algorithms in order to increase the recognition accuracy, and will extend the algorithms in order to detect and recognize fake banknotes. Moreover, it is proposed to create a virtual bank of banknotes in order to avoid the extra memory.

## Figures and Tables

**Table 1:** Banknote detection matrix of confusion, %.

Classification	Positive	Negative
	Banknote Correctly Classified	Banknote Incorrectly Classified
Positive	76.05	23.95
Negative	8.25	91.75

**Table 2:** Euro banknote recognition results for 200 real banknote images in order to calculate the hit rate of the system.

Images	Positive	Negative
200	195	5

**Table 3:** Euro banknote value recognition for a distance of 45 cm between the system and banknote in lighted indoor conditions, %.

Banknote	€5	€10	€20	€50	N/A
**€5**	100	0	0	0	0
**€10**	0	100	0	0	0
**€20**	0	0	93.3	0	6.7
**€50**	0	0	0	100	0

**Table 4:** Euro banknote recognition in an indoor environment at a distance of 38 cm, %.

Banknote	€5	€10	€20	€50	N/A
**€5**	100	0	0	0	0
**€10**	0	82.35	0	0	17.65
**€20**	0	0	100	0	0
**€50**	0	0	0	100	0
